# miR-499a inhibits the proliferation and apoptosis of prostate cancer via targeting UBE2V2

**DOI:** 10.1186/s12957-021-02371-7

**Published:** 2021-08-24

**Authors:** Yougan Chen, Fanghao Sun, Liansheng Zhang, Jian Zhou, Jianquan Hou

**Affiliations:** Department of Urology, The First Affiliated Hospital of Soochow University, Suzhou, 215006 China

**Keywords:** UBE2V2, miR-499a, Prostate cancer

## Abstract

**Background:**

Prostate cancer is one of the malignant tumors of the urinary system and ranks second among the fatal cancers in men. And with age, the incidence of prostate cancer will increase linearly.

**Methods:**

In this study, we measured the expression of Ubiquitin Conjugating Enzyme E2 V2 (UBE2V2) in prostate cancer tissues and cell lines by WB and explored the effect of UBE2V2 on the proliferation characteristics of prostate cancer by MTT and colony formation test.

**Results:**

In our research, we found that the UBE2V2 protein level in prostate cancer cell lines was significantly higher than the UBE2V2 protein level in normal prostate cells, and the mRNA expression level did not change significantly compared with normal prostate tissue cells. At the same time, we found that miR-499a combined with UBE2V2 inhibited the expression of UBE2V2 in prostate cancer cells.

**Conclusions:**

In conclusion, our results indicate that miR-499a inhibits the proliferation of human prostate cancer cells by targeting UBE2V2, which will provide a potential target for the treatment of prostate cancer.

## Background

Prostate cancer is a malignant tumor with a very high incidence of the male urinary system [1]. The disease is caused by malignant tumor lesions in the epithelium of the prostate [2]. The main population is 70–80-year-old males. The onset of prostate cancer has a great relationship with heredity. People with family genetic history have an earlier age of onset [3]. From the current point of view, there are no obvious symptoms in the early stage of prostate cancer, and it is often found in the late stage. Besides, it’s very easy to metastasize and endanger the life of the patient [4].

It is well known that the occurrence, development, and metastasis of different tumors are similar to a certain extent [5–8]. Cancer metastasis is an important cause of more than 90% of cancer-related deaths, but our understanding of the molecular mechanisms that regulate metastasis is still limited [9, 10]. On the other hand, the invasion-metastasis cascade is a multi-step cellular process involving the spread of cancer cells through the surrounding extracellular matrix, survival in the circulation and initial seeding, and then expansion in a heterogeneous microenvironment [11]. Recent evidence shows that microRNAs (miRNAs) are small non-coding RNAs that regulate various biological processes and play an important role in regulating cancer cell metastasis, tumor development, and metastasis. Numerous studies have shown that specific miRNAs highlighted by the miR-16, miR-330-5p, miR-34a, let-7, miR-10b, miR-93, and miR-200 families may act as initiating genes or inhibitors of cancer cell metastasis through a variety of mechanisms [12–14].

In the current prostate cancer (PCa) research, several types of prostates have been discovered using functional assays such as tracking cell surface markers (CD44, CD133, etc.), side populations and ethyl fluoride, and lineage tracking strategies based on reporter genes [15–17]. The cancer cells of these prostate cancer populations have been shown to have high clonality, invasiveness, and metastatic activity and have a certain resistance to castration, docetaxel, and many other therapeutic drugs [18]. However, there is still little knowledge about how cancer cells can regulate their proliferation, migration, and drug resistance through miRNAs. Ubiquitin Conjugating Enzyme E2 V2 (UBE2V2) protein is a special class in the E2 protein family. They have sequence similarity to other ubiquitin-conjugating enzymes but lack the conserved cysteine residues that are essential for the catalytic activity of E2 [19, 20]. Current research shows that the protein may be closely related to the differentiation of a variety of cells [21]. In the previous screening of miRNA libraries targeting prostate cancer cells to regulate miRNAs, we found that miR-499a is significantly under-expressed in several prostate cancer cell populations, and we have also detected abnormally low expression of miR-499a in prostate cancer tissue samples. Current research shows that mir-499a can usually inhibit the expression of proto-oncogenes in a variety of normal cell tissues and maintain the normal morphology of cells and tissues [22–24]. Based on our research, we believe that the abnormal expression of miR-499a and UBE2V2 in tissues and samples may be closely related factors affecting the occurrence and development of prostate cancer.

## Materials and methods

### Clinical sample collection

Three cases of prostate cancer tissues and paired peri-tumoral tissues were obtained from patients with prostate cancer diagnosed at The First Affiliated Hospital of Soochow University. All protocols about the use of patient samples were approved by the Medical Ethics Committee of the Affiliated Hospital of The First Affiliated Hospital of Soochow University (LS2019046).

### Cell lines and culture conditions

Human prostate cancer cell lines (PC3) were purchased from the Chinese Academy of Sciences, Shanghai Institute of Biochemistry and Cell Biology (Shanghai, China). PC3 cells were maintained in DMEM (Invitrogen) (10% FBS).

### RNA extraction and qRT-PCR

The extraction of total RNA and the analysis of qRT-PCR were performed according to the previous description. We used TRIZOL reagent (Thermofisher, USA) to extract total RNA by in cells and tissues. Taqman probes (Applied Biosystems, USA) were used to quantify miRNAs. Briefly, 1 μg of total RNA was transcribed to cDNA using AMV reverse transcriptase (Takara, Japan) and a RT primer. The reaction conditions were: 16 °C for 30 min, 42 °C for 30 min and 85 °C for 5 min. Real-time PCR was performed using a Taqman PCR kit on an Applied Biosystems 7300 sequence detection system (Applied Biosystems, USA). The reactions were performed in a 96-well plate at 95 °C for 10 min, followed by 40 cycles of 95 °C for 10 s and 60 °C for 1 min. GAPDH was used as the internal control. Primers: GAPDH: Forward 5′-GCACCGTCAAGGCTGAGAAC-3′, Reverse 5′-ATGGTGGTGAAGACGCCAGT-3′; miR-499a Forward 5′--AACAUCACAGCAAGUCUGUGCU-3′, Reverse 5′-UUAAGACUUGCAGUGAUGUUU-3′; UBE2V2 Forward 5′-CCGCTCGAGATGGCGGTCTCCACAG-3′, Reverse 5′-CGGGATCCTTACAGATCCTCTTCTGAGATG -3′.

### Western blotting analysis

The PC3 cells were washed twice with PBS (ice-cold) and centrifuged at 12,000*g* for 10 min at 4 °C; protein from tumors or cells was isolated using cell lysis buffer (Thermo Fisher, MA, USA). And then the protein was separated on a 10% SDS–PAGE and transferred to PVDF membrane. Next, membranes were incubated with 0.5% bovine serum albumin for 1 h at room temperature followed washed by PBS. Then membranes were incubated with primary antibodies (1:1000) at 4 °C overnight. It was washed and incubated in secondary antibody at room temperature for 1–2 h. Finally, the bands were evaluated on scanning densitometry through enhanced chemiluminescence (ECL, Thermo Fisher, MA, USA). GAPDH served as a loading control and protein bands were quantified using the Image J Software.

### Cell viability assay

PC3 cells were plated in 96-well plates (1 × 10^3^ cells per well). At 0, 1, 2, and 3 days, cell viability was determined using MTT. Absorbance at 570 nm of each sample was recorded.

### Plasmid construction and luciferase reporter assay

In short, the 3′-UTR sequence of UBE2V2 is searched from the NCBI [25, 26]. And the 3′-UTR of UBE2V2 that contained the presumed miR-499a binding sites (https://www.targetscan.org). We used pMIR-REPORT Luciferase NC (Ambion) to construct pMIR-UBE2V2-3′-UTR plasmid. The implementation method refers to the previous study [27, 28].

### Colony formation assay

The mixture of 5 × 10^3^ cells and 0.3% agar solution in DMEM containing 10% FBS and neomycin was poured on top of a 0.6% agar layer in six-well plates. Then the plates were maintained at 37 °C in a humid condition with 5% CO_2_ for 3 weeks. After being stained by p-iodonitrotetrazolium violet, the colonies were observed microscopically (EVOS XL Core, Thermo, USA).

### Plasmid construction and siRNA interference

UBE2V2 knockdown was accomplished by transfecting cells with siRNA. UBE2V2 and control siRNA were synthesized by Synthgene (China). The implementation method refers to the previous report [29]. Control plasmid (pCMV6) and overexpression plasmid (pCMV6-UBE2V2) came from Synthgene (China).

### Statistical analysis

The results were expressed as the mean ± standard deviation of the mean of three independent experiments. Comparisons were determined using Student’s *t* test and a *p* < 0.05 was considered statistically significant.

## Results

### UBE2V2 protein is up-regulated in prostate cancer.

**Fig. 1:**
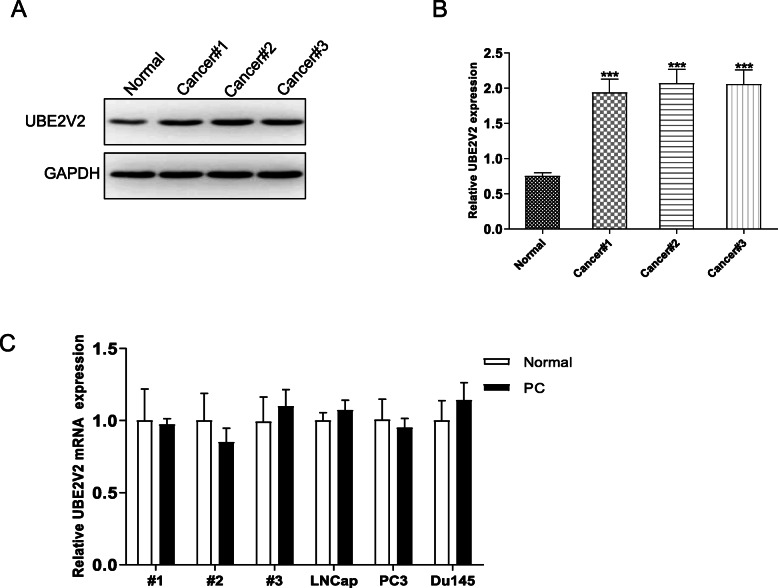
Detection of UBE2V2 protein and mRNA expression levels in prostate cancer tissues. **A** WB analysis the expression of UBE2V2 protein in 3 pairs of prostate cancer tissues and peritumoral tissues. **B** Quantify the protein bands of the UBE2V2 protein (O.D. ratio over GAPDH). **C** The mRNA expression levels of UBE2V2 in 3 pairs of prostate cancer tissues and peritumoral tissues. Data are shown as mean ± SEM (*n* = 3). Asterisks indicate significant differences from the control (*, *p* < 0.05; **, *p* < 0.01; ***, *p* < 0.001, Student’s *t* test)

In order to investigate the role of UBE2V2 on prostate cancer, 3 pairs of prostate cancer tissues and peritumoral tissues (normal) were used to measure the expression levels of UBE2V2. As shown in Fig. 1A, B, when compared with the normal tissues, the protein expression levels of UBE2V2 in prostate cancer tissues were significantly upregulated. We also used these samples to measure the mRNA levels of UBE2V2. Intriguingly, there were no striking differences between cancer and adjacent tissues in UBE2V2 mRNA levels (Fig. 1C). These results suggested that UBE2V2 may play a role in prostate cancer pathogenesis, and UBE2V2 was regulated post-transcriptionally in prostate cancer.

### UBE2V2 promotes cells proliferation in vitro.

**Fig. 2:**
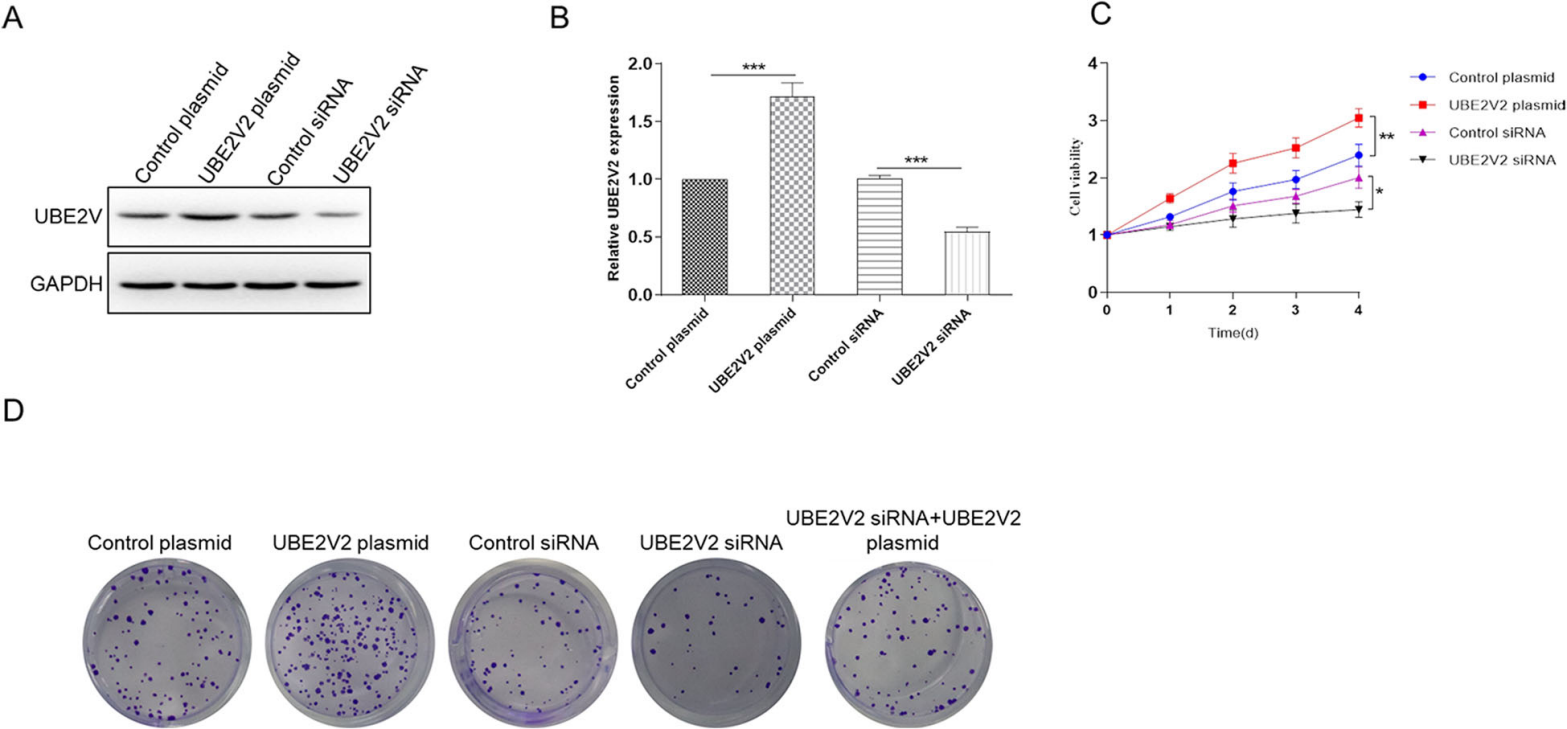
Role of UBE2V2 in the proliferation of prostate cancer cells. **A** WB analysis the expression of UBE2V2 protein in PC3 cells after transfection with control plasmid, UBE2V2 plasmid, control siRNA, or UBE2V2 siRNA. **B** Quantify the protein bands of the UBE2V2 protein (O.D. ratio over GAPDH). **C** Cell viability of PC3 cells was measured by MTT assay. PC3 cells were transfected with control plasmid, UBE2V2 plasmid, control siRNA, UBE2V2 siRNA, or co-transfected with UBE2V2 siRNA and UBE2V2 plasmid. **D** Colony formation assay of cell proliferation after PC3 cells were transfected with control plasmid, UBE2V2 plasmid, control siRNA, UBE2V2 siRNA, or co-transfected with UBE2V2 siRNA and UBE2V2 plasmid. Data are shown as mean ± SEM (*n* = 3). Asterisks indicate significant differences from the control (*, *p* < 0.05; **, *p* < 0.01; ***, *p* < 0.001, Student’s *t* test)

pCMV6 were used to construct UBE2V2 overexpression plasmid, an expression plasmid that expresses the full-length ORF of UBE2V2 without 3’-UTR. Then, we transfected the overexpressed plasmid into PC3 cells. Subsequently, we knocked down UBE2V2 via siRNA interference technology. Firstly, we detected the expression of UBE2V2 protein to confirm whether UBE2V2 was overexpressed and knocked down (Fig. 2A, B). Next, we detected the proliferation of prostate cancer cells. Compared with control group, the cell proliferation was significantly up-regulated in UBE2V2 overexpression cells. In contrast, the cell viability was significantly down-regulated in UBE2V2 knock-down cells (Fig. 2C). Finally, colony formation assay showed that the proliferation rate of PC3 cells was significantly increased following the overexpression of UBE2V2 (Fig. 2D), while the proliferation rate of PC3 cells was significantly decreased following the inhibition of UBE2V2 by siRNA. In addition, the proliferation rate of PC3 cells was rescued when co-transfected with both UBE2V2 overexpression plasmid and UBE2V2 siRNA when compared with control plasmid or control siRNA (Fig. 2C, D).

### UBE2V2 is a target gene of miR-499a

**Fig. 3:**
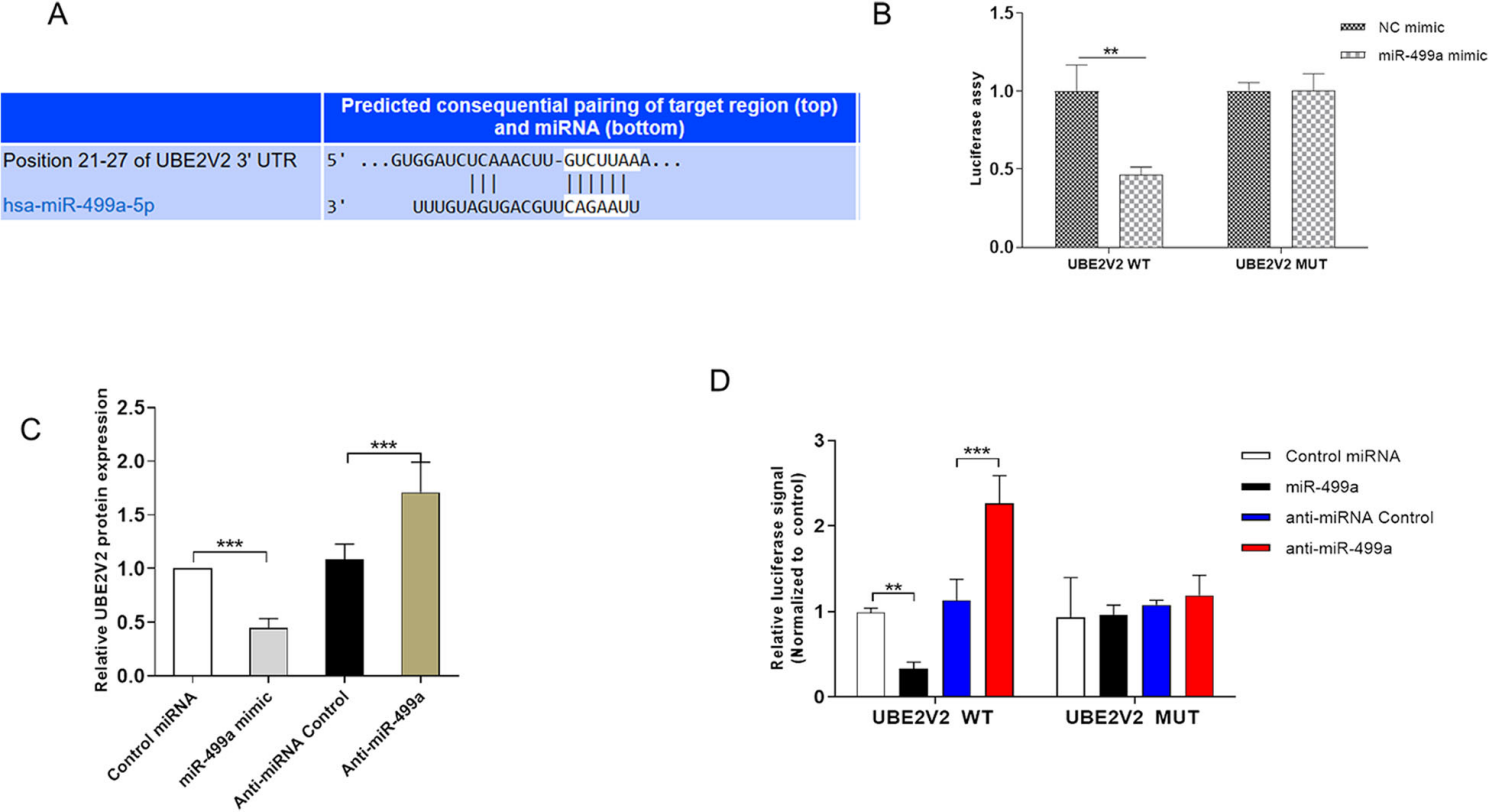
Detection of the correlation between UBE2V2 protein levels and the levels of miR-499a. **A** 3′-UTR base pairing diagram of miR-499a and UBE2V2. **B** The mRNA expression levels of *miR-499a* in 3 pairs of prostate cancer tissues and peritumoral tissues. **C** Relative expression level of UBE2V2 after transfection. **D** Luciferase assy to confirm the binding of miR-499a and UBE2V2. Data are shown as mean ± SEM (*n* = 3). Asterisks indicate significant differences from the control (*, *p* < 0.05; **, *p* < 0.01; ***, *p* < 0.001, Student’s *t* test)

As shown in Fig. 3A, the UBE2V2 gene was found to contains two putative sites of the 3′-UTR untranslated region (3′-UTR) that matched to the miR-499a seed region. And luciferase assay was used to determine the binding side of miR-499a and UBE2V2. Replacement of Guanine base with Cytosine (G to C), Cytosine bases with Guanine (C to G) or Adenine bases with Uracil (A to U) can also be used for the construction of mutant reporter. To verify whether miR-499a directly regulated UBE2V2, we detected the expression of UBE2V2 when overexpressing and knocking down miR-499a. As shown in Fig. 3C, the expression level of UBE2VE was significantly up-regulated when transfected with miR-499a Anti-miR-499a. Nevertheless, the expression level of UBE2V2 was significantly down-regulated when transfected with miR-499a mimics.

We further set up the luciferase reporter plasmid (containing the wild-type (WT) and mutation-type (MUT) 3′-UTR) of the target gene UBE2V2 by luciferase reporter NC. Compared with control groups, WT reporter activity was predominantly decreased in PC3 cells when transfected with miR-499a mimics (Fig. 3D), while WT reporter activity was strikingly increased in PC3 cells when transfected with miR-499a inhibitor. In sharp contrast to WT reporter activity, the transfection of miR-499a mimics or miR-499a inhibitor did not affect the activity of MUT reporter activity. All in all, these studies suggested that miR-499a negatively regulated UBE2V2 expression by directly binding to the 3′-UTR region of UBE2V2 in prostate cancer.

### miR-499a suppresses cell proliferation via regulating UBE2V2 in vitro

**Fig. 4:**
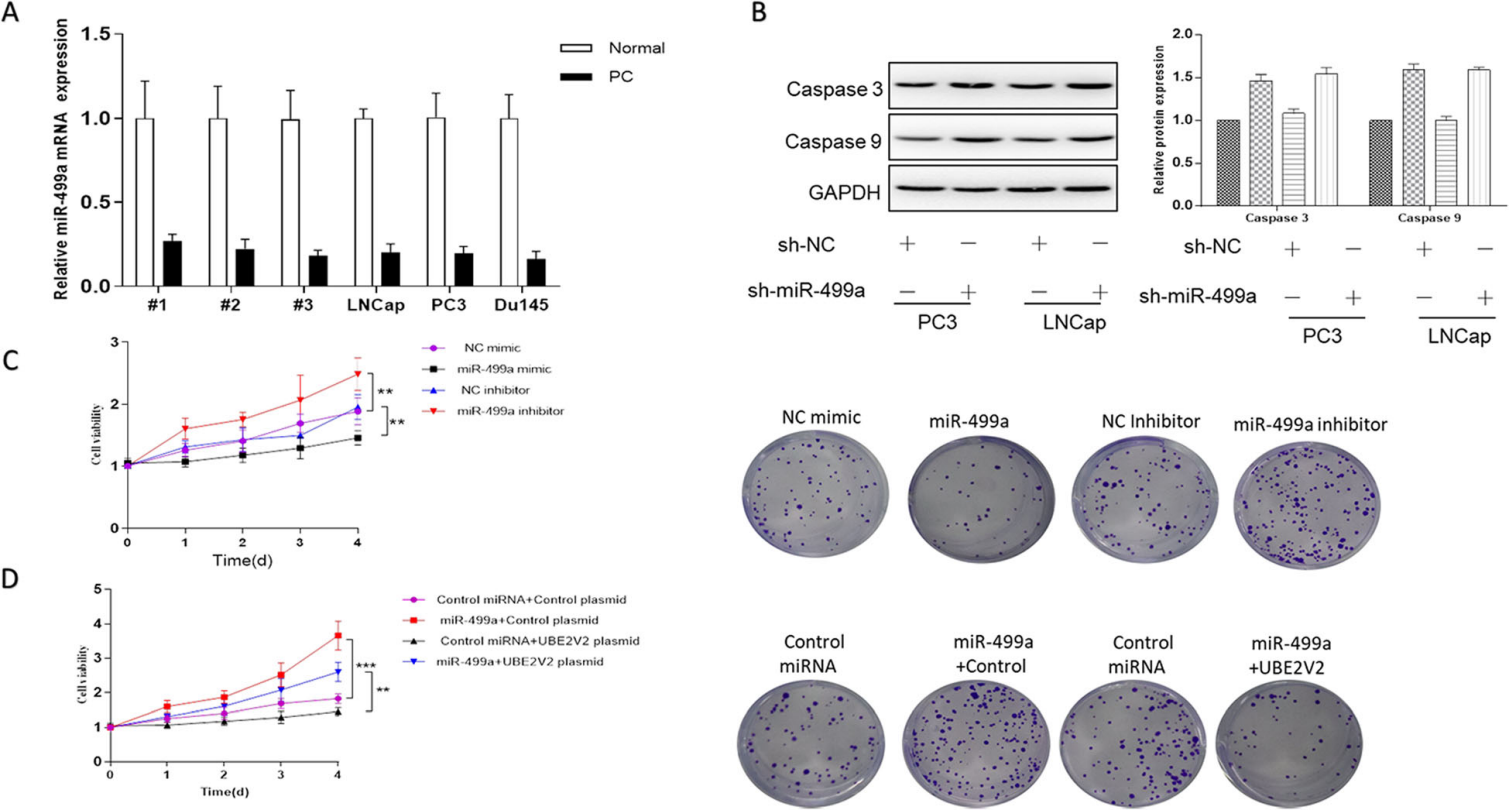
miR-499a directly regulates gene expression of its target UBE2V2. **A** The mRNA expression levels of miR-499a in all samples and cancer cell lines. **B** WB analysis the expression of Cas-9 and Cas-3 protein in PC3 and LNCap cells after transfection with control miRNA, miR-499a. **C** Cell viability and colon formation assay after transfected with control palsmid, miR-499a plasmid, and UBE2V2 plasmid. **D** Cell viability and Colony formation assay of cell proliferation after PC3 cells were transfected with NC mimic, miR-499a mimic, NC inhibitor, miR-499a inhibitor, control miRNA + control plasmid, miR-499a + control plasmid, control miRNA + UBE2V2 plasmid, miR-499a + UBE2V2 plasmid. Data are shown as mean ± SEM (*n* = 3). Asterisks indicate significant differences from the control (*, *p* < 0.05; **, *p* < 0.01; ***, *p* < 0.001, Student’s *t* test)

In Fig. 3B, we found that the expression of miR-499a was significantly down-regulated in prostate cancer tissues. Therefore, we tried to explore the role of miR-499a-UBE2V2 pathway in prostate cancer. As shown in Fig. 4A, overexpression of miR-499a led to a significant decrease in cell proliferation while knockdown of miR-499a led to a significant increase in cell proliferation. Intriguingly, co-transfection of miR-499a mimics and UBE2V2 plasmid into PC3 cells was able to rescue the effect of miR-499a in cell proliferation (Fig. 4C). In order to study the effect of miR-499a on the apoptosis of prostate cancer cells, we used WB to detect the expression of apoptosis-related proteins in PC3 and LNCap groups after transfection.

As we can see (Fig. 4B), the expression of apoptosis-related proteins Cas-9 and Cas-3 in the two groups of cell lines after miR-499a transfection was significantly higher than that of the blank group, indicating that the apoptosis of prostate cancer cells in the two groups increased after miR-499a was increased.

At the same time, we also carried out a clone formation assay (Fig. 4D), Our experimental results show that increasing the expression of miR-499a in PC3 cells can significantly inhibit the proliferation of PC3 cells. Similarly, to study the effects of miR-499a and UBE2V2 on cell proliferation and apoptosis, we increased the expression of miR-499a and UBE2V2 in PC3 cells, respectively. We found that increasing the expression of miR-499a can offset the overexpression of UBE2V2 in PC3 cells. The cell proliferation caused by miR-499a can inhibit the proliferation and migration of prostate cancer cells by inhibiting the expression of UBE2V2 in prostate cancer cells (Fig. 4E). These results suggested that miR-499a inhibited the proliferation of cancer cells by targeting UBE2V2 in prostate cancer.

## Discussion and conclusion

It is well known that the age of prostate cancer patients is usually older than 65 years and the prognosis is generally poor. The malignant proliferation of tumor cells is the main factor affecting the prognosis and threatens the lives of many patients [30]. In the past few decades, many genes have been found to be involved in the regulation of the proliferation of prostate cancer, but the function of UBE2V2 in the proliferation of prostate cancer has not been reported. In this study, we determined that UBE2V2 is a pathogenic factor for prostate cancer.

Previous studies have shown that UBE2V2 is related to the occurrence and development of various tumors. The study by Hua zhi-dan et al. has shown that the expression of UBE2V2 in lung adenocarcinoma is positively correlated with PD-L 1[21]. And the expression of UBE2V2 is negatively correlated with the severity of lung adenocarcinoma. The study by Oakman et al. in molecular breast cancer showed that UBE2V2 is an important marker of the patient's prognosis in breast cancer tissues [31]. In this study, we found that the expression of UBE2V2 protein was significantly increased in prostate cancer tissues, and the overexpression of UBE2V2 significantly promoted the proliferation and migration of cancer cells, while knocking down UBE2V2 can inhibit the proliferation and migration of cancer cells. In addition, we also detected the UBE2V2 mRNA level, which is inconsistent with the protein level results, indicating that there is a post-transcriptional regulatory mechanism in UBE2V2.

Through previous studies on the mechanism of cancer cell proliferation, we found that miRNA usually regulates the expression of target genes at the post-transcriptional level [32]. To confirm our hypothesis, we identified miR-499a as a candidate miRNA through bioinformatics. Previous studies have shown that, under normal circumstances, miR-499a can inhibit the expression of proto-oncogene in tissues and can inhibit tumor growth in many cancers. The study of Gu Xiaobin et al. showed that miR-499a can affect the sensitivity of breast cancer chemotherapy by regulating the epithelial-mesenchymal transition [22]; the study of Heshan et al. in lung cancer showed that low expression of miR-499a can promote lung fibrosis, Seriously affect the patient's recovery [33]. In this study, we found that miR-499a was significantly down-regulated in prostate cancer tissue, and its level was negatively correlated with the expression level of UBE2V2 (Figs. 1 and 2). Subsequently, we used the luciferase reporter gene NC to establish a luciferase reporter plasmid for the target gene UBE2V2. We further proved the regulatory relationship between miR-499a and UBE2V2 (Fig. 3). Finally, we clarified that miR-499a promotes the proliferation of prostate cancer cells in vivo and in vitro by targeting UBE2V2 (Fig. 4). All these results indicate that the tumor suppressor function of miR-499a in prostate cancer is mainly achieved by targeting UBE2V2.

## Conclusion

All in all, we have identified the pathogenic factors and new regulatory networks of prostate cancer. This research may find a new method for the treatment of prostate cancer in the future.

## Data Availability

The datasets used and/or analyzed during the current study are available from the corresponding author on reasonable request.
